# Does Using Multiple Tobacco Products Open the Door to Mental Health Disorders? A Cross-Sectional Study among Tunisian High School Students

**DOI:** 10.1192/j.eurpsy.2026.10888

**Published:** 2026-07-31

**Authors:** S. Ben Fredj, R. Ghammam, A. Skhiri, N. Zammit, O. Ben Saad, F. Chouikha, A. Chouchen, J. Maatoug, I. Harrabi

**Affiliations:** 1 Department of Preventive Medicine and Epidemiology, “LR19SP03”, University Hospital Farhat Hached; 2 Faculty of Medicine of Sousse, University of Sousse; 3 Department of Occupational Medicine, “LR19SP03”, University Hospital Farhat Hached , Sousse, Tunisia; 4 Community and Preventive Medicine Department, Primary health Care Corporation, Doha, Qatar

## Abstract

**Introduction:**

Tobacco use remains a major public health concern among adolescents, with growing evidence linking it not only to physical harm but also to mental health challenges. While e-cigarettes and other tobacco products are increasingly popular among youth, less is known about how patterns of multiple product use relate to anxiety, depression, self-esteem, and digital behaviors.

**Objectives:**

To determine the association between the use of many tobacco products and mental health disorders among Tunisian High School Students.

**Methods:**

A cross-sectional study was carried out in the urban area of Sousse, Tunisia, during the 2018/2019 school year. A representative sample of high school students attending public institutions was recruited. Data were collected using a self-administered structured questionnaire that captured sociodemographic information and mental health outcomes (including depression, anxiety, self-esteem, alexithymia, and problematic use of video games and Facebook), assessed through validated Arabic scales. Statistical analyses were performed using SPSS software, version 20. We created clustering groups by adding each one-tobacco product (Cigarette, e-cigarette , and Waterpipe) to obtain 4 aggregations from 0 to 3.

**Results:**

A total of 1,342 students participated (63.2% female; mean age 17.5 ± 1.4 years). The prevalence of tobacco use was 16.5%. We found that 83.5% of the studied population were nontobacco users, 2.7% had the maximum number of poly-tobacco use, 8.1% were single users, and 5.7% were dual users. The proportion of anxiety decreased significantly (0.002) with the number of tobacco products used. The high school students who did not use any tobacco products were more likely to have anxiety, with 86.9% compared to students using 3 products (50%). Yet, students who used 2 or 3 tobacco products presented a problematic use of videogames (33.3% and 27.6% respectively), significantly higher than those who used none or one product (10.5% and 21.7% respectively). Users of three products were more likely to be depressed, have low self-esteem, and have a problematic use of Facebook. However, the associations were nonsignificant

**Conclusions:**

Using multiple tobacco products among adolescents was associated with disorders in mental health and digital behaviors. While causality cannot be inferred from this cross-sectional study, the findings underscore the need for preventive programs that address both substance use and emerging digital risks. Future longitudinal research is warranted to clarify these relationships and guide effective interventions.

**Disclosure of Interest:**

None Declared

